# Predictability of Invisalign^®^ Clear Aligners Using OrthoPulse^®^: A Retrospective Study

**DOI:** 10.3390/dj10120229

**Published:** 2022-12-06

**Authors:** Luca Levrini, Andrea Carganico, Alessandro Deppieri, Stefano Saran, Salvatore Bocchieri, Piero Antonio Zecca, Sara Bertini, Anna D’Apote, Marzia Segù

**Affiliations:** 1 Department of Human Sciences and Innovation for the Territory, University of Insubria, 21100 Varese, Italy; 2 Department of Medicine and Surgery, University of Insubria, 21100 Varese, Italy; 3 Independent Researcher, GOT, 10121 Torino, Italy; 4 Department of Medicine and Surgery, University of Parma, 43126 Parma, Italy

**Keywords:** clear aligner appliances, orthodontic treatment, photobiomodulation, dentistry

## Abstract

This preliminary retrospective study evaluates how effective the OrthoPulse^®^ (Biolux Technology, Austria) is in increasing the predictability of orthodontic treatment in patients treated with Invisalign^®^ clear aligners (Align Technology Inc., Tempe, AZ, USA). A group of 376 patients were treated with Invisalign^®^ orthodontic clear aligners in association with an OrthoPulse^®^. The OrthoPulse^®^ was prescribed for 10 min a day for the entire duration of the orthodontic treatment. The OrthoPulse^®^ App remotely tracked the percentage compliance of each patient. The number of aligners planned with the ClinCheck software at the beginning of the treatment and the number of total aligners (including the adjunctive aligners) used to finish the treatment were then considered. After applying inclusion/exclusion criteria, a total of 40 patients remained in the study and were compared with a control group of 40 patients with the same characteristics as the study group. A statistical analysis was carried out to investigate whether using OrthoPulse^®^ led to a statistical reduction in the number of adjunctive aligners, thus leading to a more accurate prediction of the treatment. The statistical analysis showed that patients who used OrthoPulse^®^ needed fewer finishing aligners and a greater predictability of the treatment was obtained. In fact, in the treated group the average number of additional aligners represented 66.5% of the initial aligners, whereas in the control group 103.4% of the initially planned aligners were needed. In conclusion, in patients treated with clear aligners, OrthoPulse^®^ would appear to increase the predictability of orthodontic treatment with clear aligners, thus reducing the number of finishing phase requirements.

## 1. Introduction

Photobiomodulation (PBM), or LLLT (low level laser therapy), was introduced in the 1960s by Endre Mester, who conducted experiments with lasers on mice skin and other animal models. In these latter studies, no changes were found in tumor volume; but in the treated group, the hair was found to grow more rapidly. This and many other experiments led to PBM being used in photomedicine [1].

Photobiomodulation uses a variety of light sources with varying properties, such as lasers and LEDs (e.g., wavelength, output power, continuous-wave or pulsed operation modes and pulse parameters). Longer wavelengths (between 800 and 900 nm) and larger output powers (up to 100 mW) have been more popular in recent years for therapeutic devices, particularly to enable deeper tissue penetration. Several devices have obtained marketing clearance from the FDA; however, none of them has been expressly approved for use in dentistry. Lasers differ in terms, for example, of the beam, depth of penetration, wavelength, length of the “on time” when pulsed, and effects on the eye.

The best compromise between power output (500 mW) and safety is thought to be achieved by PBM lasers. Substituent diode lasers are frequently used in PBM lasers. There are two main types: gallium-aluminum-arsenide (GaAlA), which emits invisible light between 750 and 910 nm, and indium-gallium-aluminum-phosphide (InGaAIP), which emits visible light between 630 and 680 nm. Other wavelengths are also possible. The use of surgical hard and soft tissue lasers in non-contact and defocused modes at low energy output levels (500 mW) can also have positive benefits for PBM [2].

With lasers, combined wavelengths are difficult to replicate, and the small beam width makes treating large regions challenging. Consequently, PBM devices also include a non-laser LED group with a range of wavelengths. Infrared to near-infrared light from LED arrays is produced at ideal wavelengths and energy densities. The more recent LEDs, which were initially created by Harry Whelan and his team at the NASA Space Medicine Laboratory in 1998, have less divergence, far greater and more consistent output powers, and quasi-monochromaticity, in which nearly all the photons are at the specified wavelength. Another characteristic of LEDs is “photon interference”, which is caused by multiple intersections of the LED beam from different LEDs. Due to this and the physical forward and backward scattering properties of ionizing radiation (IR) and non ionizing radiation, target cells placed further within the tissue have exceptionally strong photon intensities [3].

**Figure 1:**
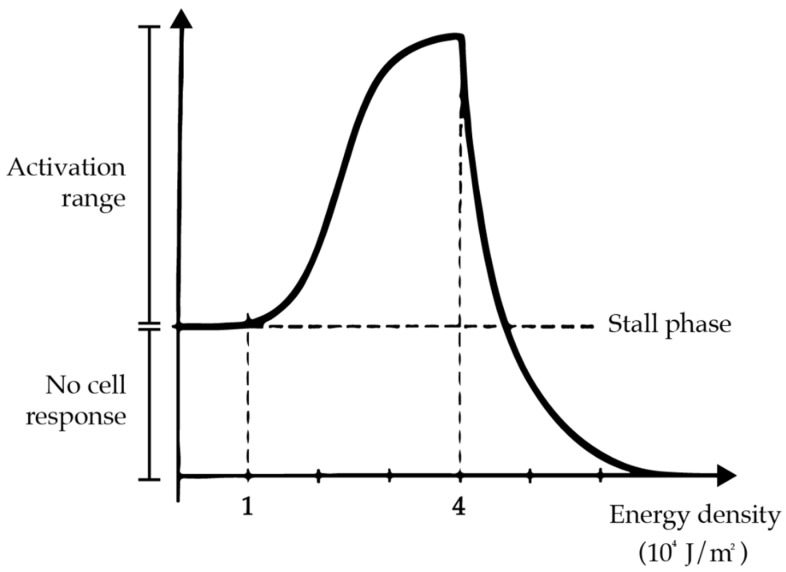
The Arndt-Shulz law.

The biological operating principle of PBM is based on the Arndt Schulz law. According to this law, a biphasic dose–response relationship can be observed [4]: low doses of energy produce no visible effects, but excess doses produce an inhibitory effect on cellular function [5] (Figure 1).

Therapeutic results can in fact be achieved only by remaining within certain dosages, known as the therapeutic window [6,7].

The specific biochemical process underlying the therapeutic effects of PBM is still unclear., According to a growing body of research, the largest effect is thought to be the stimulation of mitochondrial cytochromes, which then triggers secondary cell-signaling pathways. Hemoglobin, myoglobin, and cytocrome c oxidase (CCO) are the three main photoacceptor molecules found in mammalian tissues.

Cytocrome c oxidase is responsible for producing and metabolizing energy. This was validated when it was discovered that the absorption spectra for CCO in various oxidation states were strikingly similar to the action spectra for biological reactions to light.

**Figure 2:**
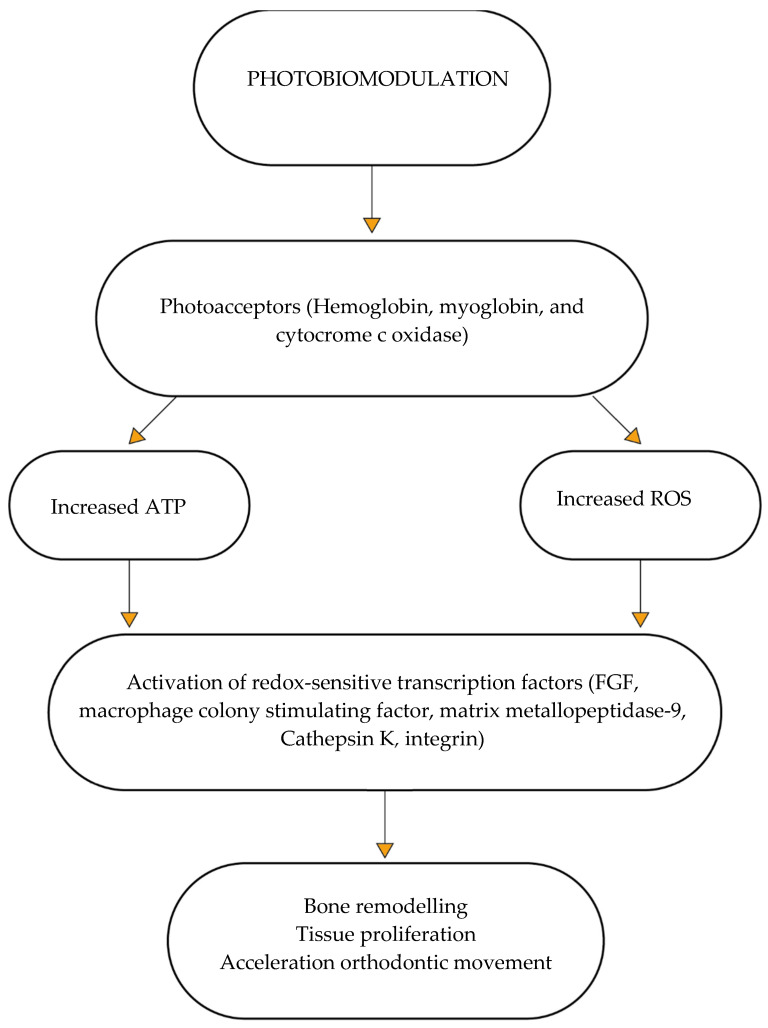
Photobiomodulation mechanism of action.

Nitric oxide (NO) attaches to CCO in the mitochondria, particularly in stressed or hypoxic cells, by competitively dislodging oxygen, preventing cellular respiration, and thus reducing ATP synthesis. Photobiomodulation may function by reversing the mitochondrial inhibition of respiration and the production of reactive oxygen species by photodissociating NO from CCO (ROS). Redox-sensitive transcription factors are activated as a result of this change in the total redox potential of the cell, which favors higher oxidation and ROS production. Additionally, NO is photodissociated from other intracellular reserves, such as nitrosylated myoglobin and hemoglobin, leading to vasodilation [8]. This therapy’s low doses of photonic radiation thus stimulate cells through molecular and chemical mechanisms, causing low amounts of reactive oxygen species and adenosine triphosphate (ATP) synthesis as well as photon absorption in the mitochondria (ROS). By encouraging the proliferation and differentiation of several cell lineages such as osteoblasts, osteoclasts, fibroblasts, and periodontal ligament cells, this anabolic impact further accelerates the movement of the teeth and has a stimulatory effect on bone remodeling, collagen formation, and revascularization (Figure 2). This process is aided by the expression of the molecules’ basic fibroblast growth factor, macrophage colony stimulating factor, c-fms, tartrate-resistant acid phosphatase, matrix metallopeptidase-9, Cathepsin K, and integrin. The effectiveness of photobiomodulation therapy in lowering pain and accelerating orthodontic tooth movement has been the subject of numerous studies in the past. However, the uniquely uniform properties of the many types of lasers employed and the variations in laser settings have led to contradictory results.

In order to obtain the biological effects required, four parameters, in addition to the wavelength, need to be taken into account: the energy required for activation {(*E*/*a*)*act*}, the total irradiation time (Δ*t_tot_*), the beam cross-section (*a*), and the light intensity threshold [*I*_0_]. All these parameters are part of the LILAB (low-intensity-laser-activated-biostimulation) equation:

(1) $${\left(E/a\right)}_{act}=I_{stim}\cdot \Delta t_{tot}$$

where *I_stim_* is the intensity of energy needed to overcome the intensity threshold *I*_0_. The PBM works when *I_stim_* is greater than *I*_0_, thus biologically affecting the irradiated tissue [5].

Orthodontic treatments are used both for young and older patients. In young patients, the need for an aesthetic is less than in older patients. In addition, in young patients the kind of treatment requested can be different, in particular when they are still growing. In these cases, functional therapy is sometimes required aimed at modulating the development of the bones rather than the movement of the teeth. Even young patients, when they have finished growing, need treatment to resolve possible dental malocclusion.

Thanks to this greater use and the demand for more aesthetic and comfortable solutions than fixed orthodontics, orthodontic techniques are changing in order to meet patient needs [9,10,11]. In 1998, Align Technology Inc. developed the Invisalign^®^ technique, which uses removable pre-printed polyurethane aligners, as an alternative to the classic fixed orthodontics. Almost 10 million patients have already been treated using this method.

With the Invisalign^®^ technique, the number of clear aligners is planned for each patient at the beginning of the treatment using ClinCheck. With this, clinicians can plan the treatment digitally and set the desired outcome from the beginning. Nevertheless, a second phase of treatment is generally necessary, requiring an additional number of aligners to achieve the desired result. The need for a second phase is due to the lack of predictability of the treatment planned with the ClinCheck. In fact, the predictability of clear aligner treatments is challenging and affects the outcome [12]. The accuracy in the predictability of dental movement is about 41% [13], and around 70−80% of clinicians need to make corrections during the therapeutic process [14].

**Figure 3:**
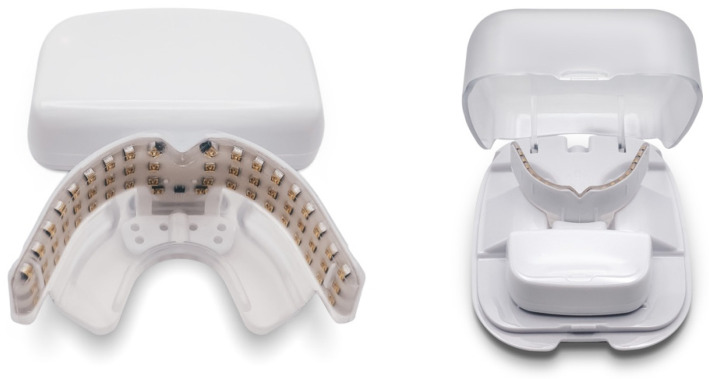
The OrthoPulse.

To ensure the correct application of this orthodontic technique, some devices have been developed to help both the clinician and the patient to obtain the result desired. Among these, OrthoPulse^®^ (OP), uses the principles of PBM with the emission of low intensity light (850 nm +/− 80 nm) to facilitate bone remodeling (Figure 3).

The purpose of our study was to evaluate a possible correlation between PBM, obtained with the use of OP, and the predictability of orthodontic treatment with Invisalign^®^ clear aligners.

## 2. Materials and Methods

This study was carried out in accordance with the ethical standards established in the 1975 Helsinki Declaration and the Ethics Committee of the Ospedale di Circolo. Signed informed consent was obtained for all the patients. All the data collected were anonymous; all participants provided informed consent and agreed to the privacy policy for the protection of personal data.

In this retrospective study, 376 patients were selected from a database of patients treated by the same group of orthodontists. All the subjects had undergone orthodontic treatment with Invisalign^®^ (Align Technology Inc., Tempe, AZ, USA) clear aligners in association with an OrthoPulse^®^ (Biolux Technology, Absdorf, Austria), prescribed for 10 min a day for the duration of the treatment. The OP device delivers PBM to the tissues of the oral cavity by emitting infrared light that maintains a continuous wavelength of 850 nm and a power of 90 mW/cm^2^, using about 54 LEDs placed 5 mm apart in two banks of 3 × 9 components implanted in a flexible silicon matrix.

The compliance rate of the device (expressed as the ratio between the prescribed and the real use as a percentage) was assessed using the OrthoPulse^®^ App. To achieve maximum compliance (100%), patients have to have used OP for at least 10 min a day for the entire duration of orthodontic treatment, synchronizing the application each time the device is used. An incorrect use of the device or mistakes in the synchronization process can result in lower patient compliance.

The sample inclusion criteria for this study were: patients in permanent dentition, a minimum of 25% of the compliance rate for the use of the OP device (tracked with the OP app), suitable diagnostic records, and the absence of periodontal disease and tooth decay.

Patients with a DMFT (Decayed, Missing and Filled Teeth) index higher than 3, insufficient or inconsistent data, and systemic or metabolic disease that might have modified the compliance rate were excluded from the study.

A total of 40 patients were selected for the study group (Group 1). The control group consisted of another 40 patients, with the same characteristics, treated by the same team of orthodontists with Invisalign^®^ clear aligners but without the OP device (Group 2).

For the statistical analysis, the following parameters were considered for each patient: age, gender, number of aligners planned by the CC, number of finishing aligners, and number of total aligners used. From these data, to assess the predictability of orthodontic movement, the percentage was calculated using the number of total aligners compared to the number of aligners predicted by the initial CC.

The statistical analysis was carried out using SPSS v. 22.0 (SPSS Inc., Chicago, IL, USA). The Shapiro–Wilk test was used to check whether data were normally distributed. All parameters were calculated as average and standard deviations. The Student T-test for independent samples was run to report any significant changes between measurements between Groups 1 and 2. The statistical significance was set at *p* < 0.05.

## 3. Results

A sample size of 40 subjects for both Group 1 and Group 2 was required to detect an effect size of 0.05 (with a statistical power of 0.8). The results are summarized in Table 1. Group 1 consisted of 40 patients: 14 males and 26 females, with an average age of 34.1. The average number of aligners scheduled for these patients at the beginning of the treatment was 29.5 and the average number of finishing aligners was 19.6. The final number of aligners was thus 49.2. The percentage variation between the aligners planned with ClinCheck at the beginning of the treatment and the number of aligners used to finish the treatment was 66.5%. The average compliance rate was 79.7% (SD 20.3%).

The control group (Group 2) consisted of 40 patients, 14 males and 26 females, with an average age of 32.5. The average number of aligners planned for these patients at the beginning of the treatment was 26.5, the number of finishing aligners was 27.4, while the average number of total aligners was 54. The percentage variation between the aligners planned with the ClinCheck at the beginning of the treatment and the number of aligners used to finish the treatment was 103.4%. There was thus a greater need in Group 2 patients for finishing aligners; in fact, the percentage variation was 36.8%, which was higher than in Group 1. The T-test confirmed the statistical significance of these results (*p* < 0.05).

## 4. Discussion

**Figure 4:**
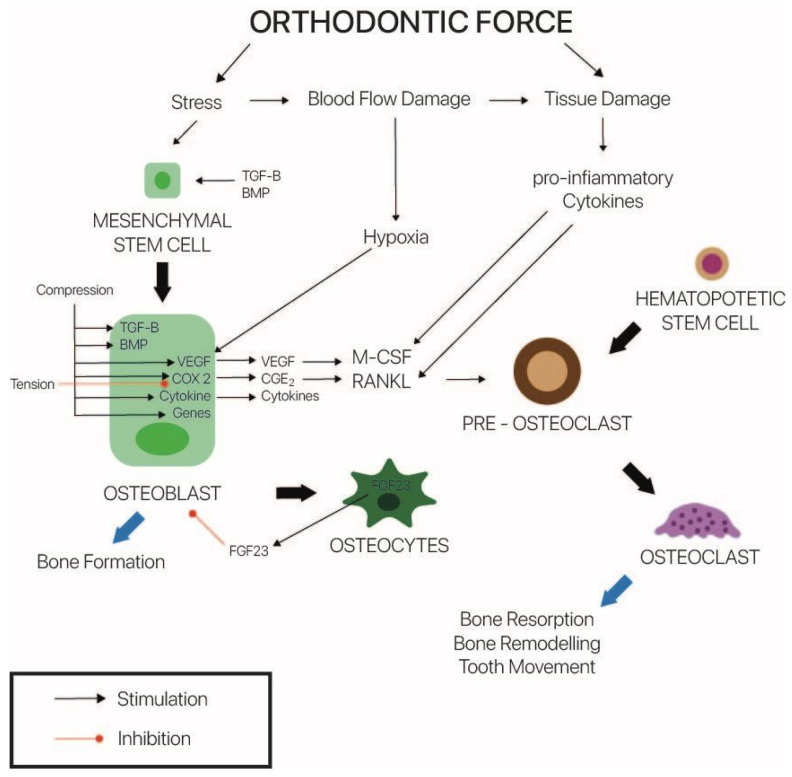
The orthodontic movement.

The intricate process of orthodontic tooth movement involves the remodeling of periodontal and dental structures. Both the periodontal ligament and the alveolar bone, which are two very distinct tissues with varied remodeling potentials, need to undergo biochemical changes during this process (Figure 4).

Accelerating orthodontic movement is a key trend in modern orthodontics and there is a growing interest in this field. The shorter treatment duration is definitely preferable from the perspective of both the patient and the practitioner as it can decrease the risk of decalcification, dental caries, gingival inflammation, root resorption, and patient burnout. Numerous methods have been studied to speed up tooth movement, including surgical procedures, the local injection of cellular mediators, pulse electro-magnetic fields, mechanical vibration, low-level laser therapy (LLLT), and light-emitting diode LED-mediated photobiomodulation (PBM) [15].

The surgical methods include corticotomy, osteotomy, periodontal ligament distraction, or micro osteoperforations [16]. Pharmacologically, protaglandins, corticosteroids, parathormone (PTH), nitric oxide, and osteocalcin accelerate the orthodontic movement [17]. Physical-mechanical methods such as vibration, electromagnetic stimulation, and photobiomodulation (PBM) are currently being developed.

Our aim was to assess any correlation between PBM, obtained with the use of OP, and the predictability of orthodontic treatment with Invisalign^®^ clear aligners. We investigated whether using an OP would reduce the number of finishing aligners. This device delivers PBM to the tissues of the oral cavity by emitting infrared light that maintains a continuous wavelength of 850 nm and a power of 90 mW/cm^2^.

Photobiomodulation in the medical field has rapidly increased [2,18,19,20,21,22] thanks to its ability to influence different types of tissues. In fact, infrared light stimulates mitochondrial activity by promoting the production of ATP, the reduction of oxidative stress, and cell proliferation. Specifically, PBM produces targeted effects depending on the tissue irradiated. At a vascular level, an increase in endothelial cells, the stimulation of angiogenesis, and increased vasodilation is observed. On the other hand, for non-mineralized tissues, there is an increase in collagen type 1, fibroblasts, and fibronectin. In mineralized tissues, there is an increase in the number of osteoclasts and osteocalcin and also an increase in osteoclastic activity and alkaline phosphatases (ALP). Finally, there are also changes in the immune system due to the increase in transforming growth factors (TGFs) and basic fibroblast growth factors (bFGF). There is also an increase in RANK, RANK-L, and IGF-1 receptors and an increase in interleukin (IL-1β) levels [4,8,23,24,25,26,27].

Photobiomodulation is widely used in the medical field for various disorders: from acute pain [19] to rheumatoid arthritis [20,21], from post-traumatic edema [2,18] to Bell’s palsy [22], and the prevention of bisphosphonate-induced osteonecrosis [28]. Photobiomodulation is used in the dental field thanks to its interaction with bone tissue [29]. Photobiomodulation is also employed to assess the predictability of treatments or the acceleration of orthodontic movement even though in the literature there is low evidence about that [8,23,24,26,27]. Photobiomodulation can increase cell growth factors and promote the synthesis of enzymes such as metalloproteinases (MMP)-9 and cathepsin K. The proliferation of these factors plays an essential role in bone remodeling and could consequently accelerate orthodontic movement [30]. Photobiomodulation could thus also be used in other orthodontic areas, such as the acceleration of dental movement. According to other studies, the interaction between light and tissues at the mitochondrial level could be useful not only in terms of predictability, but also in terms of treatment duration [30,31,32,33,34].

These properties are also useful in orthodontics; hence, the development of the OP device. The distinctive aspect of this study was the use of a portable LED device instead of a clinical setting-based laser light source. This PBM delivery approach is appealing since it does not require costly laser equipment, and it involves less chair time and less operator training.

In the orthodontic field, PBM is already used in various techniques. Nimeri et al. investigated the changes in root morphology in a group of 20 patients, using cone beam computed tomography to detect root resorption. The association of PBM was found not to cause greater root resorption compared to traditional orthodontic treatment [35]. Güray and Yüksel investigated the effect of PBM on the rate of canine distalization. In this randomized controlled clinical trial, 30 subjects were treated using OP for five minutes/day. An acceleration of 33% in the canine distalization rate was observed in the patients using PBM [36]. Al-Dboush et al. investigated the effect of PBM and low-intensity pulsed ultrasound (LIPUS) to accelerate orthodontic movement in a sample of 84 subjects treated with clear aligners. The use of PBM led to an average reduction of 26.6% in treatment time, while low-intensity pulsed ultrasound led to an average reduction of 26% [37].

According to the manufacturer, OP uses low-intensity near-infrared light technology to stimulate the bone and facilitate tooth movement, leading to a reduction in treatment time. The device uses a constant emission of continuous infrared light at 850 nm and a power density of 90 mW/cm^2^ thanks to 54 LEDs placed apart in two banks of 3 × 9 components implanted in a flexible material, and it is prescribed for 10 min a day for the entire duration of the treatment. Also, the compliance percentage can be monitored by the mobile OP app.

Today, there is an increasing demand for aesthetic treatments that have a lower impact on the social lives of young people. However, there is a greater need for aesthetic therapy in adults [38]. In fact, until new approaches with a low impact on the patient’s physical appearance have been developed, many patients reject orthodontic therapy and prefer not to treat the malocclusion. Aesthetical and comfortable devices have also started to be adopted in the interceptive approach. The number of patients treated with clear aligners is growing rapidly. Compared with traditional fixed appliances, clear aligners offer a more aesthetic and comfortable alternative [39]. In addition, the use of removable appliances reduces the negative effects of fixed orthodontics on the periodontal tissues and allows better oral hygiene [9].

The CC enables both clinicians and patients to plan the final result of the orthodontic treatment, providing the number of aligners needed to achieve the final result. Nevertheless, the clinician may need to make some changes and adjustments requiring finishing sets of aligners and prolonging the duration of the treatment [13]. Although the CC helps the clinician to plan the orthodontic treatment and improve the interaction with the patient [40], it does not often provide precise data on the number of total aligners needed to complete the therapy [13,41,42].

According to the literature, the predictability provided by supporting instruments, such as the CC, is far from 100%. Approximately 70–80% of patients require additional aligners over and above those predetermined by the CC in order to complete the treatment. Moreover, the percentage accuracy in terms of the reliability of the CC ranges from 41% to 72%, which is closely related to the results obtained in the sample in this study, and above all to the type of movement analyzed [13,14,42].

The predictability of clear aligner treatments is strongly influenced by the compliance of patients, which must be constantly motivated and instructed. Regarding this aspect, in recent years social media are gaining more and more importance, and they could lead to significant improvements in knowledge while being a useful aid to verbal motivation [43]. Rossini et al. showed that the movement with the lowest predictability is extrusion (approx. 30%), followed by rotation (approx. 36%), whereas the most predictable movement is distalization of the upper molar (approx. 88%). Other movements such as intrusion (46%), alignment (78%), mesiodistal tipping (46%), and lingual vestibule (49%) were analyzed on the basis of the data in the literature [11].

The percentage variations are due to the biomechanical difficulty of aligners in performing specific dental movements. The more difficult a movement is, the less predictable it will be and consequently the number of aligners will be greater.

In this study, the specific types of movements were not evaluated; however, the number of aligners was assessed in patients treated with the OP device compared to a sample group.

From our study, we can therefore state that the use of OP during orthodontic treatment increases the predictability of the treatment plan developed with the CC, thus making PBM a valid method to achieve better results in terms of the predictability of the orthodontic movement.

In Group 1, the average compliance rate of the OP device was 79.7%. A statistically significant reduction in the number of finishing aligners was observed in Group 1. In fact, in Group 1 the average number of additional aligners represented 66.55% of the initial aligners, while in Group 2 103.4% of the initially planned aligners were needed.

This study is not without limitations, and the main issue is the relatively small size of the sample, which limits the study’s power to adequately assess the several factors involved. Moreover, the compliance rate obtained with the monitoring app may have been influenced by a lack of perfect synchronization between the device and the patient’s smartphone. It is thus possible that low compliance rates were due to a lack of synchronization rather than the scarce use of the OP. Furthermore, within the two groups, specific orthodontic movements planned by the CC were not considered. This could have led to more specific results regarding the predictability of tooth movement.

Another aspect regards the wavelength used by the device.

Although the OP uses a wavelength of 850 nm, no guidelines have been published to show which wavelength is the most effective. Some studies have been conducted using wavelengths ranging from 630 nm to 940 nm [8,32,44,45]. However, wavelengths between 700 nm and 850 nm deeply penetrate the tissues, with two peaks around 725 nm and 810 nm [46]. Other studies have shown that the wavelengths that deliver concrete orthodontic effects are 630 nm, 660 nm, 830 nm, and 850 nm [47,48]. The wavelength of the light, the thickness of the target tissue, and the degree of the keratinization and pigmentation of the oral mucosa may all have an impact on the depth of penetration. There is an estimated 7% decrease in laser energy provided for every millimeter increase in alveolar bone thickness, according to reports in the literature [49].

In their review of the literature, AlShahrani et al. found that 80% of the experiments used lasers with a wavelength of between 750 and 900 nm, and that the greater the wavelength, the less tooth movement. This is consistent with Arntz Schutz’s law, which states that laser therapy exhibits inhibitory effects at longer wavelengths.

Regarding energy density, most investigations used a value of approximately 4.2–8 J/cm^2^, and only a small number of these showed a favorable effect. Even denser amounts did not produce any appreciable effects. The conclusion is that an energy density of less than 4.2 J/cm^2^ can also have a major impact because, in some circumstances, lower densities can have biological consequences on irradiated tissue.

It has also been reported that different power levels had different effects on tooth movement: 20–100 mW produced favorable results, while 200 mW had no impact [6].

Today, devices capable of inducing PBM have an LED component in addition to the laser component. When LED arrays emit light at the best wavelengths, it can reach a depth of about 23 cm in skin and tissue. Due to the use of a variety of wavelengths, a wider beam width, and the ability to treat greater regions with less heat output, LEDs are therefore an effective substitute for lasers. In contrast, a lot of LED devices are often relatively low powered (about 5–15 mW), and a lot of this energy is scattered, necessitating longer treatment periods.

The use of organic LEDs in PBM devices is a future development. These LEDs have an organic film that emits light in response to an electric current as the emissive electroluminescent layer.

Further research is required to determine which wavelength, energy density, and power output give the best results.

## 5. Conclusions

The PBM delivered by the OP device might help to increase the predictability of orthodontic treatment with clear aligners, thus reducing the number of finishing aligners and the duration of the treatment. The low intensity light delivered by the OP device could improve the amount of dental movement planned with the ClinCheck, resulting in a more predictable and precise orthodontic treatment. Using OrthoPulse would seem to be a good option in order to reduce the time of the treatment and accelerate the orthodontic movement. Further research is needed to confirm these findings.

## Figures and Tables

**Table 1:** Key results.

	Compliance	Planned Aligners	Finishing Aligners	Total Aligners	% Variation
Group 1	79.75% ± 0.20	29.52 ± 12.06	19.65 ± 18.00	49.17 ± 20.75	66.55%
Group 2	N.A.	26.57 ± 12.32	27.48 ± 20.32	54.05 ± 26.93	103.42%

## Data Availability

The data that support the findings of this study are available from the corresponding author (L.L.) upon reasonable request.
